# Identifying the barriers to kidney transplantation for patients in rural and remote areas: a scoping review

**DOI:** 10.1007/s40620-023-01755-0

**Published:** 2023-09-01

**Authors:** Tara K. Watters, Beverley D. Glass, Andrew J. Mallett

**Affiliations:** 1 College of Medicine and Dentistry, James Cook University, Townsville, QLD Australia; 2 Department of Renal Medicine, Cairns Hospital, PO Box 902, Cairns, QLD 4870 Australia; 3 Department of Renal Medicine, Townsville University Hospital, Townsville, QLD Australia; 4 Institute for Molecular Bioscience, The University of Queensland, Brisbane, QLD Australia

**Keywords:** Chronic kidney disease, Kidney transplant, Rural and remote, Indigenous health, Barriers to healthcare

## Abstract

**Background:**

Populations in rural and remote areas have higher rates of chronic kidney disease and kidney failure than those in urban or metropolitan areas, and mortality rates for chronic kidney disease are almost twice as high in remote areas compared to major cities. Despite this, patients residing in regional, rural, or remote areas are less likely to be wait-listed for or receive a kidney transplant. The objective of this scoping review is to identify specific barriers to kidney transplantation for adult patients residing in rural and remote areas from the perspectives of health professionals and patients/carers.

**Methods:**

Studies were identified through database (MEDLINE, CINAHL, Emcare, Scopus) searches and assessed against inclusion criteria to determine eligibility. A descriptive content analysis was undertaken to identify and describe barriers as key themes.

**Results:**

The 24 selected studies included both quantitative (*n* = 5) and qualitative (*n* = 19) methodologies. In studies conducted in health professional populations (*n* = 10) the most prevalent themes identified were perceived social and cultural issues (80%), burden of travel and distance from treatment (60%), and system-level factors as barriers (60%). In patient/carer populations (*n* = 14), the most prevalent themes were limited understanding of illness and treatment options (71%), dislocation from family and support network (71%), and physical and psychosocial effects of treatment (71%).

**Conclusions:**

Patients in regional, rural, and remote areas face many additional barriers to kidney transplantation, which are predominantly associated with the need to travel or relocate to access required medical testing and transplantation facilities.

**Graphical abstract:**

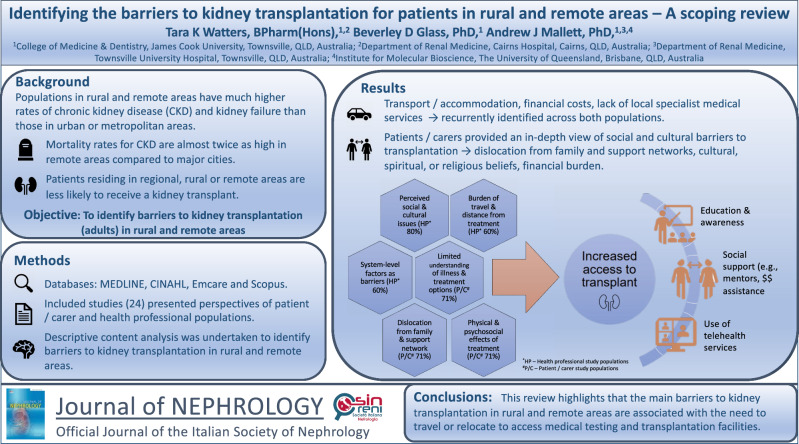

**Supplementary Information:**

The online version contains supplementary material available at 10.1007/s40620-023-01755-0.

## Introduction

The prevalence and financial burden of kidney failure is increasing worldwide with an estimated global chronic kidney disease (CKD) prevalence of > 10% [1]. Kidney transplantation is considered the gold standard kidney replacement therapy, as it offers significant cost saving benefits for health care systems [2] as well as better quality of life and improved survival for patients compared to dialysis [3]. However, variation exists between access to, and use of, kidney transplantation worldwide. In Australia, patients must have commenced on dialysis to be eligible for deceased donor transplantation, with pre-emptive transplant only possible if a suitable living donor is available [4]. Statistics indicate growing wait-list numbers and increasing wait times for kidney transplantation, with annual transplant rates representing only a fraction of wait-listed patients [5–7].

The demand for deceased donor organs exceeds current supply, and this is especially relevant given up to 80% of donated kidneys are from deceased donors [5, 6]. It is of concern that in Australia there has also been an abrupt increase in deceased donor kidney non-utilisation, which does not appear to be fully explained by changes in recorded donor characteristics [8]. There is significant geographical variability in deceased donor transplantation rates across different states, territories, and provinces within countries [9–11]. This is likely attributable to multiple factors, such as differences in recipient eligibility criteria and variations in usage of marginal organs between transplant centres, and differences in supply and demand of deceased donor kidneys between states [9].

Living donor kidney transplantation is associated with longer graft and patient survival, and enables pre-emptive transplantation prior to commencement of dialysis [4, 12]. However, rates of living donor kidney transplantation have plateaued or even significantly dropped in some countries, despite the overall increase in total number of transplants occurring each year [5–7]. It is also recognised that certain patient populations, such as Indigenous patients and lower socioeconomic groups, find it more difficult to access living donor kidney transplantation compared to deceased donor transplantation [12–14].

Populations in rural and remote areas have much higher rates of CKD and kidney failure than those in urban areas, and mortality rates for CKD are almost twice as high in remote areas when compared to the major cities [15–17]. Despite this, it is well documented that CKD patients residing outside of urban areas are less likely to access specialist kidney services for treatment or receive the recommended screening or education about CKD and the available options for kidney replacement therapy [18, 19]. Patients residing in rural, or remote areas are also far less likely to be wait-listed for or receive a kidney transplant [20–22]. Indigenous peoples in Australia, New Zealand, Canada and the United States are considered high-risk groups with regard to CKD as they are more than twice as likely to progress to kidney failure than non-Indigenous peoples [23], particularly if they reside in a rural or remote area [24]. They are also less likely to be deemed eligible for kidney transplantation, and those who are eligible experience longer delays to activation on the wait-list [23, 25].

Lack of access or delay to transplantation has both resource and quality of life implications, and evidence indicates that for kidney transplant recipients, a longer time spent on dialysis prior to transplant is associated with worse long-term outcomes and overall survival [26]. In Australia, the average annual cost to the economy in 2021 was estimated to be more than $182,000 AUD per person living with kidney failure, mostly attributable to the high cost associated with dialysis [27]. Given the relative shortage of donor organs, barriers to kidney transplantation for all kidney failure patients are vast and may include medical, surgical or psychosocial ineligibility [28]. Given the lower rates of kidney transplantation in rural and remote populations around the world, it is likely that additional barriers exist for this patient population [20].

The objective of this scoping review is to investigate the extent of current literature identifying the specific barriers to kidney transplantation for adult patients residing in rural and remote areas.

## Materials and methods

To investigate the extent of current literature on barriers to kidney transplantation in rural and remote areas, a scoping review methodology was chosen. The framework developed by Arksey and O’Malley was used to comprehensively review the literature in five stages: (1) identifying the research question, (2) identifying relevant studies, (3) selecting the relevant studies, (4) charting the data, and (5) collating, summarizing and reporting the results [29]. This scoping review was conducted in accordance with the guidelines published by the Joanna Briggs Institute (JBI) [30] and is reported in accordance with the Preferred Reporting Items for Systematic Reviews and Meta-analyses (PRISMA) extension for scoping reviews checklist [31].

### Search strategy and information sources

An initial limited search of relevant databases was undertaken to identify articles related to the research topic. With the assistance of an academic librarian, an analysis of keywords and index terms used to describe identified articles was undertaken to develop a full search strategy, which was adapted for each database. The first author searched the following online databases (21st July, 2022 and 20th December, 2022): MEDLINE (Ovid), CINAHL Complete, Emcare on Ovid, and Scopus. The search strategy did not include any limitations such as study design, language or year of publication. The final search strategies are provided in Online Resource 1. The reference lists of all identified reports and articles were also searched to identify any additional relevant studies.

### Study identification and selection

Eligibility criteria for included studies were developed collaboratively among all authors and was directed by the review objective, with the aim being to include only studies that would provide rich and in-depth data relevant to the specified participants, concept and context [30].

Eligible international studies included those focused on identifying barriers to kidney transplantation in rural, remote or Indigenous adult populations, as well as those investigating barriers to organ donation as part of the transplantation process. Studies investigating barriers to all or other modalities of kidney replacement therapy (e.g., dialysis) were included only if kidney transplantation was specifically mentioned in the results. Studies addressing barriers to all or other forms of organ transplantation were not included. Eligible studies presented data collected directly from relevant health professionals, patients, or their caregivers. Studies presenting database or registry data or medical record review alone were ineligible. Review articles or those not published in English were not included.

Citations for all identified articles were collated and imported into the EndNote database management system and duplicate records removed. The first author screened the title and abstract of all articles against the inclusion criteria, and for potentially relevant sources the full text data was retrieved. The full text articles were reviewed by all authors and assessed in detail against the inclusion criteria to determine final eligibility. Any disagreements that arose regarding eligibility of sources were discussed among all authors, until a consensus was reached. Reasons for exclusion of sources were recorded.

### Data extraction and synthesis

A data extraction tool was developed collaboratively by all authors to collect and present relevant data. The first author extracted data from included studies such as country of origin and year of publication, clinical aspect of focus and objectives of study, study population and sample size, and methodology used (including validation of methods).

In order to identify and describe key concepts in the findings, included studies were grouped according to the study participants: health professionals or patients/family/carers, and a descriptive content analysis was undertaken. The results and findings of each study were inductively coded line-by-line by the first author (using NVivo software) to develop initial descriptive themes specific to each study population. This method was chosen to enable direct comparison between studies undertaken within the same population and identify translation across both populations. The key findings for each study are presented as the list of descriptive themes and concepts identified, which were reviewed and discussed by all authors. Consistent with the objectives of this review and guidance for conduct of scoping reviews, the methodological quality of individual studies was not appraised [30].

## Results

**Fig. 1:**
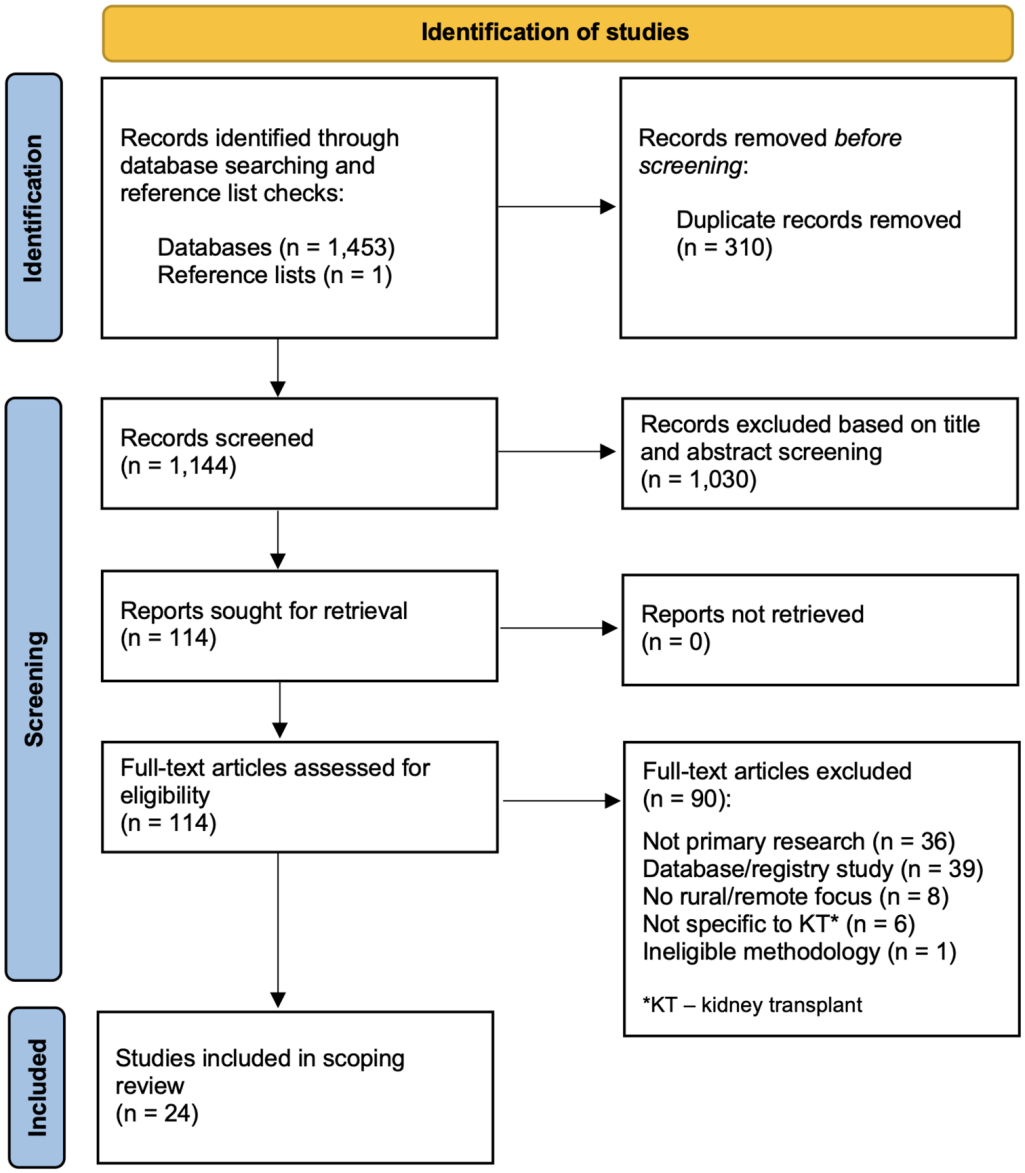
Identification of studies for inclusion in scoping review

Through database searches and manual searching of reference lists a total of 1454 citations were identified (Fig. 1). Following removal of 310 duplicates, the title and abstract of 1144 citations were screened and 1,030 excluded. Full text articles for 114 citations were retrieved and assessed in detail against inclusion criteria, with 90 full text articles excluded. The remaining 24 studies were included in this scoping review.

Included studies were published between 1995 and 2022, with most (19/24) published in the last 10 years and conducted in Australia and/or New Zealand. A quantitative methodology (survey) was used for data collection in only 21% (5/24) of included studies [32–36], with a qualitative methodology (interviews, focus group discussions, workshop discussions) used in 79% (19/24) of studies [37–55]. Study participants included both health professionals involved in the kidney transplantation (or donation) process (10/24), as well as CKD patients, their family members or carers, and potential or actual kidney donors (14/24). Study characteristics, key themes identified and summarised recommendations across included studies are provided in Online Resource 2. Themes identified across included studies are outlined below, with frequency of themes identified across separate study participant groups presented in Table 1.

**Table 1:** Frequency of identified themes across studies

Health professional perspective themes	% Total (*n* = 10)	Patient/Carer perspective themes	% Total (*n* = 14)
Communication barriers	40% (4)	Communication barriers	64% (9)
Burden of travel and distance from treatment	60% (6)	Burden of travel and distance from treatment	57% (8)
Fear of negative outcomes	50% (5)	Fear of negative outcomes	36% (5)
Perceived limited understanding of illness and treatment options	50% (5)	Limited understanding of illness and treatment options	71% (10)
Social and cultural issues			
Perceived social and cultural issues	80% (8)	Indigenous-specific cultural responsibilities	29% (4)
		Religion, spirituality and cultural beliefs	50% (7)
		Dislocation from family and support network	71% (10)
		Experiences of racism and cultural bias	14% (2)
		Financial burden of treatment	43% (6)
		Involvement of family and community in treatment decisions	29% (4)
System-level factors			
System-level factors as barriers	60% (6)	Lack of continuity of care	29% (4)
		Impact of late presentation or diagnosis	21% (3)
Adherence issues			
Pre-transplant adherence and engagement	50% (5)	Non-adherence or inability to engage with treatment	36% (5)
Poor definition and assessment of adherence	20% (2)		
Physical and psychosocial wellbeing			
Safeguarding psychological wellbeing	40% (4)	Physical and psychosocial effects of treatment	71% (10)
Justifying living kidney donor sacrifice	20% (2)		
Motivation for transplant			
Advocating for transplant as a treatment option	10% (1)	Motivation for transplant	36% (5)
Transplantation processes			
Balancing benefit to patient versus maximising utility of donor kidneys	40% (4)		
Barriers to facilitating organ and tissue donation	10% (1)	Perceptions around organ donation	14% (2)
Shortage of donor kidneys	40% (4)	Hesitancy to accept a donated kidney	29% (4)
Medical comorbidities as a barrier to transplantation	40% (4)	Tedious pre-transplant work-up	36% (5)

### Communication barriers

This theme was identified across 64% (9/14) of studies carried out in patient/carer populations, and 40% (4/10) in health professional populations. For patients, communication barriers were associated with differences in language, literacy, values, and preferred communication styles between patients and health providers [39, 40, 52]. Use of medical jargon, overly complex English, specialists speaking too fast or being overly assertive and a perceived reluctance of specialists to spend time speaking with patients contributed to difficulty understanding information regarding kidney transplant [39, 42, 52]. Patients also reported feeling intimidated in large, unfamiliar and busy institutional settings and even those actively seeking information did not feel comfortable questioning staff or seeking clarification [42, 52, 55]. One Indigenous participant noted “When I first came in with kidney failure … I didn’t really get much information at all. It could have been much better than it was… Now it’s 2 years later and I’m just starting to find out about transplant… I don’t know anything about it, or how people get on the list” [42].

Health professionals reported difficulties communicating with non-English speaking patients or those with low health literacy, particularly in helping them to understand the complex process and relay complicated information to carers and families regarding kidney transplantation [36, 45]. There were concerns around the emotional elements and accuracy of using interpreters to relay information, and some nephrologists felt that non-English speaking patients were not referred for transplantation because of perceived difficulties in navigating these communication barriers [45]. The lack of culturally appropriate transplant education materials for Indigenous patients was also of concern for health professionals, who did not feel equipped to provide appropriate education to patients and carers with different cultural understandings of health [36, 50].

### Burden of travel and distance from treatment

This theme was identified across 57% (8/14) of studies carried out in patient / carer populations, and 60% (6/10) in health professional populations. For patients and carers, increased distance from transplant centre resulted in significant financial burden and logistical difficulties associated with travel and transportation, housing, and temporary accommodation [43, 46, 47, 49, 51, 52, 54]. One participant said “We lived there for 30 years and unfortunately when I developed renal failure, we realised we’d have to be near a larger hospital. So, we had to sell off our farm. We left the town where all our friends were and moved, 180 kms away” [51]. One survey study investigating costs incurred by living kidney donors found the highest direct costs were related to travel and accommodation [32]. In many cases patients and their families were left to arrange travel and accommodation themselves, with very little support or resources available to reduce this burden. Access to specialist or allied health professionals (such as dieticians, exercise physiologists and other support professionals) and medical testing required as part of transplantation work-up is limited in rural and remote areas, meaning potential living donors and recipients must also travel to access these services [46, 47, 51, 54].

Health professionals identified issues with being able to provide adequate care, education, and information to remote patients [37, 45, 50]. They also acknowledged the financial and logistical issues faced by patients in having to travel or relocate to comply with work-up requirements and/or receive a kidney transplant [37, 38, 44, 50]. Lack of access to services (dentistry, allied health, vascular and bariatric surgery) required to complete transplantation work-up was identified as a major barrier for these patients [50]. One survey study found that nephrologists practising in rural settings were more likely to consider complexities of caring for the post-transplant patient and scarcity of transplant centres in the area in the decision not to refer patients for transplant [33].

### Fear of negative outcomes

This theme was identified across 36% (5/14) of studies carried out in patient/carer populations, and 50% (5/10) in health professional populations. For patients and carers this was not only in relation to the process of receiving a kidney transplant [46], but also potential negative outcomes for living donors, particularly if the donor was a family member [42]. Patients acknowledged that a lack of shared knowledge about the transplant process feeds into this fear, for both them and their families [42]. One participant admitted “I put off going for transplant two times when I got the call, because I had heard from other community members how scary it was” [46]. Fear around competency of care received in rural or remote centres was also raised [51].

For health professionals, the perception that kidney transplant outcomes are relatively poor in Indigenous patients certainly contributed to fear around negative outcomes and hesitancy to refer this population for transplantation [38]. In general, fear around potential negative outcomes was related more to “high risk” candidates, however this included fear around damaging the nephrologists’ own professional reputation as well as the survival rates for the transplanting centre [45, 53]. One survey study found that younger nephrologists or nephrology trainees, and those with fewer years in practice were significantly more likely to perceive an increased risk of kidney failure for living donors and significantly less willing to recommend living donor kidney transplantation when diabetes was a factor [35]. It was also thought that patients seeing others with poor transplant outcomes may contribute to their own fear around pursuing transplantation [44].

### Limited understanding of illness and treatment options

This theme was identified across 71% (10/14) of studies carried out in patient / carer populations, and 50% (5/10) in health professional populations. For Indigenous patients and carers this was identified as a major barrier to transplantation, and often linked to difficulties in communicating with health professionals [39, 40, 55]. In other instances, it was related to variations in religious, spiritual, or cultural beliefs [41]. There was a lack of understanding around all aspects of transplantation, including eligibility criteria (for both donors and recipients), how to (or who can) initiate the process, wait-list processes, the transplantation procedure itself, and potential post-transplant complications [42, 46, 48, 49, 55]. One participant recalled “They just told me that I had to lose weight to stay on the transplant list, not how much weight or why just that I had to” [46]. Patients residing outside of urban areas were also less likely to receive supplementary information about transplant, such as videos or pamphlets, from their nephrologist [43].

Patients’ lack of understanding of the transplantation process was identified as being a major barrier to transplantation by nephrologists [44, 45]. One survey study found that nephrologists practising in rural settings were more likely to consider a patients’ limited education as a reason not to refer them for a kidney transplant [33]. It was also felt by health professionals that lack of understanding of their illness and treatment options was also a major reason for patients to decline transplant as a treatment option [44, 50].

### Social and cultural issues

Compared to the health professional cohorts, the patient and carer study participants provided more in-depth data regarding social and cultural issues; therefore numerous subthemes were identified across the studies. Dislocation from family and support networks was the most prevalent of these, identified across 71% (10/14) of studies. For patients forced to relocate to receive dialysis, being able to return home to family, community and country was a common motivation for transplantation [39, 41, 42, 46]. However, patients and carers also experienced significant distress due to prolonged periods of separation and isolation associated with travel or temporary relocation required to undergo mandatory testing and medical procedures for transplant work-up, or to receive the actual transplant [42, 46, 49, 51, 52, 54, 55]. Financial burden of treatment was another prevalent theme, identified across 43% (6/14) of studies. One survey study investigating costs incurred by living kidney donors found total direct and indirect costs averaged $8,932 AUD per donor, and 10% of donors incurred costs above $15,000 AUD [32]. One participant recalled trying to manage the expenses associated with transplant work-up, saying “How do I spread the tests out and get everything done as you need to be able to fit it into my budget. So, well I’ll get that done this month and then I’ll get something else done the next month. Um, because that was the only way that I could afford to pay for it. But that means you waiting longer to get all the assessments done. Well, you’re not even on the list yet, so it’s just putting more wait time on” [51]. Other themes identified in the findings of the patient/carer studies included: Religion, spirituality and cultural beliefs (50% (7/14)), involvement of family and community in treatment decisions (29% (4/14)), Indigenous-specific cultural responsibilities (29% (4/14)), and experiences of racism and cultural bias (14% (2/14)).

The most prevalent of all the health professional related themes was Social and cultural issues, which was identified across 80% (8/10) of studies carried out in this population. For Indigenous patients, their culture contributed to the perception of them being “high risk” transplant candidates and less likely to engage with and maintain treatment regimens [37, 38, 41]. Functional status and issues around social support were more likely to be considered when determining eligibility for transplant by rural nephrologists [44]. Social and cultural factors such as financial hardship, lack of support and complex family dynamics were also of particular concern to nephrologists in determining eligibility for kidney transplantation [45, 50]. In fact, one study found the most commonly cited patient-related reason considered in transplant referral was inadequate social support [33]. Another study also found that clinicians’ culture and religion significantly influenced their practices in initiating organ donation within the emergency department [36].

### System-level factors

For patient/carer cohorts, system level factors were identified as either: Impact of late presentation of diagnosis (21% (3/14)), or lack of continuity of care (29% (4/14)). Patients reported that late diagnosis contributed to emotional distress making it much more difficult to adjust to their diagnosis and to make informed choices regarding treatment options [39, 40, 46]. Patients and caregivers reported receiving conflicting information from different health professionals and variability in treatment, which affected communication with, and trust in, health professionals [49, 51, 52, 54]. One participant noted “As a patient we’ve got complex needs. You’ve not just got one thing you’ve got multiple. You want to be within that same health service” [51].

This theme was identified across 60% (6/10) of studies conducted in health professionals. Complexity of health systems and differences in transplantation protocols and guidelines between transplant centres, extent of pre-transplant work-up and inefficiency coordinating assessments, and inadequate resourcing were some of the barriers identified by health professionals [33, 34, 37, 45, 50, 53]. Lack of autonomy for the referring nephrologist and concerns around preserving the reputation of the transplant centre were also mentioned [45, 53].

### Adherence (also referred to as “compliance”) issues

For patient/carer cohorts, non-adherence or inability to engage with treatment was identified across 36% (5/14) of studies. Various reasons were cited as contributing factors to this, such as having to care for sick or dependent children or family members, social and cultural responsibilities, feeling uncomfortable in the hospital environment, issues with transport or accommodation, and feelings of mistrust, anger or frustration towards the healthcare system [39–41, 55]. One participant explained “I’ve just really stopped going to most appointments, I mean what’s the point, all that travel and then its 15 min and their not really doing anything, changing my pills, but the end result will be the same” [54].

For health professionals, this theme was identified as: Pre-transplant adherence and engagement (50% (5/10)), and poor definition and assessment of adherence (20% (2/10)). There were mixed views on the use of pre-transplant adherence as in indicator of post-transplant adherence, and whilst some nephrologists consider pre-transplant non-adherence as a barrier to transplantation, others do not [38, 41, 45, 53]. One survey study did however find that nephrologist recommendation for transplantation was significantly more likely for patients who were described as “compliant” with treatment [34].

### Physical and psychosocial wellbeing

For patient/carer cohorts, physical and psychosocial effects of treatment was a theme identified across 71% (10/14) of studies. The mental, physical and emotional stress that patients and carers experienced throughout the various stages of their treatment journey was widely documented, however some also felt that this was largely unacknowledged by health care staff [39–42, 46, 49, 51, 52, 54, 55]. One participant highlighted the importance of access to social support, saying “Just to have somebody to kind of vent to, to work out is this just the process or do I need more support? You know, am I coping or not coping?” [51].

For health professionals, safeguarding psychological wellbeing (40% (4/10)) and justifying living kidney donor sacrifice (20% (2/10)) were the themes identified across studies. Some nephrologists were concerned with referring patients for transplantation that may not be able to cope with potential psychological challenges, whereas others felt compelled to refer patients to provide a sense of hope [45, 53]. The importance of respecting a patients’ decision not to accept living donation to preserve psychosocial wellbeing and confidentiality was also discussed [45, 50]. In the case of living donor kidney transplantation there was also significant concern expressed regarding potential risks to the donor [35].

### Transplantation processes

For patients and carers tedious pre-transplant work-up (36% (5/14)) [43, 46, 51, 54, 55], hesitancy to accept a donated kidney (29% (4/14)) [41–43, 55], and perceptions around organ donation (14% (2/14)) [41, 48] were the themes identified.

For health professionals, shortage of donor kidneys (40% (4/10)) and balancing benefit to patient versus maximising utility of donor kidneys (40% (4/10)) were two of the most commonly identified themes. These often appeared together, as it is primarily the scarcity of donor organs that drives tension between clinician’s responsibilities to their individual patient and ensuring equitable access to transplant, and their perceived responsibility to manage organ distribution wisely [38, 44, 45, 53]. Medical comorbidities as a barrier to transplantation (40% 4/10)) [34, 44, 45, 53] and barriers to facilitating organ and tissue donation (10% (1/10) [36] were the other themes identified.

### Motivation for transplant

For patients and carers motivation for transplant was identified across 36% (5/14) of studies. In most instances the main motivation was related to being able to return home or be with their family, in what was felt to be returning to a “normal” life where they would be independent and free to travel and work again [41–43, 46, 51].

In only one study (10%) did nephrologists mention the importance of advocating for transplant as a treatment option for their patients given the better outcomes [45].

## Discussion

This scoping review focused on identifying and summarising the reported perspectives of CKD patients, their caregivers and relevant health professionals to identify barriers to kidney transplantation for patients residing in rural and remote areas. Many barriers identified arise from the need to travel to access medical testing required as part of the work-up process, and/or to receive the actual transplantation surgery. Issues around transportation and accommodation, financial costs associated with travel and medical tests required, and lack of locally available specialist medical services were recurrently identified in both patient/carer and health professional populations. Likewise, communication barriers were similarly described across both patient/carer and health professional populations. However, many of the themes identified across both study populations were weighted differently in terms of importance to each specific population, and also described differently from each perspective. This review is the first to compare the perspectives of patient / carer populations with those of the treating health professionals with regard to barriers to kidney transplantation in rural and remote areas.

Limited education and understanding of transplantation as a treatment option, whilst identified across both populations, was described more often by patient/carer populations and clearly presented a major barrier to initiating discussions or making an informed decision around kidney transplant as a treatment option. Health professionals were able to identify basic social and cultural issues such as financial difficulties, lack of social support and cultural differences as barriers to transplantation. However, patient/carer populations provided a much more in-depth view into this theme, which resulted in several more specific barriers being identified. Dislocation from their family or support network was a major social and cultural barrier to transplantation, as were religious, cultural, and spiritual beliefs and financial burden of treatment. Whilst the physical and psychosocial effects of treatment was clearly a major barrier from the perspective of the patients/carers, this was not as much of a concern for health professionals. Similar barriers were identified in a recently conducted systematic review that looked at access to all forms of kidney replacement therapy in rural communities [56]. Patient and caregiver populations from included studies in this review also identified barriers associated with lack of education and information around available treatment options, the toll of separation from family and country, the guilt and worry associated with treatment, as well as the financial burden of travel [56].

Unsurprisingly, system-level factors as barriers to transplantation was identified as a major barrier by health professionals and focused primarily on resourcing issues or disparities between health systems and transplantation protocols. Patient/carer populations however did not report system-level factors as often, consistent with the findings of the review by Scholes-Robertson et al. [56]. Whilst pre-transplant adherence issues were identified as a barrier to transplantation by health professionals, the patient/carer populations gave an in-depth view into factors they feel contribute to non-adherence or inability to engage with treatment, such as confusion, frustration or mistrust of the health system, and other cultural beliefs or responsibilities. Again, these themes have also been identified by patient/carer populations in existing literature [56]. Fear of negative outcomes was identified as a barrier by both populations, however for health professionals this extended to their own professional reputations as well as that of the transplantation centre they represented. As expected, balancing the competing principles of achieving the best outcome for their patient versus maximising the utility of donor organs was a barrier identified only by the health professional populations.

It is important to also consider the potential relationships and interaction between the identified barriers to kidney transplantation for this patient population. While undertaking this review it became evident that the identified barriers were often related, with one barrier leading to, influencing, or exacerbating another. For example, in many cases it seemed that dislocation from their family and support network often exacerbated the psychosocial burden of treatment for patients, whilst also impacting their ability to understand and make informed decisions around transplantation as a treatment option. The relationships among identified barriers has also been documented in existing literature [56].

Overall, the barriers to kidney transplantation for rural and remote patients identified in this review are consistent with those identified in existing literature (not eligible for inclusion in this review). These barriers are not unique to kidney transplantation specifically, with the financial and time burden associated with travelling, psychosocial and emotional issues, carer burden, and lack of both financial and psychosocial support being identified as barriers for rural and remote patients across numerous solid organ transplants [57]. Other research looking specifically at barriers to kidney transplantation for Indigenous populations across Australia, United States, Canada, and New Zealand highlighted similar issues, with particular emphasis on cultural and family considerations, communication barriers and religion and spirituality as major barriers for this vulnerable population [12, 25, 58]. Globally, the financial burden associated with kidney transplantation is also well documented, particularly as a barrier to living donor kidney transplantation [59], and even in Australia where residents have access to government-funded health care, the indirect costs associated with living donor kidney transplantation continue to present a significant barrier [14]. Documented increased rates of deceased donor kidney non-utilisation [8] along with geographical disparities in deceased donor transplant rates within countries [9] support the findings in this review with regard to health professionals’ views on differences in transplantation protocols and guidelines among transplant centres.

**Fig. 2:**
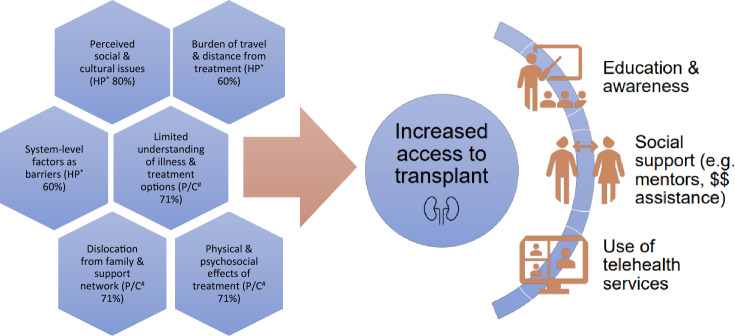
Commonly identified barriers to transplantation and recommended strategies. ^*^HP Health professional study populations. ^#^*P/C* Patient/carer study populations

Whilst none of the included studies actually evaluated interventions or solutions to address the identified barriers to transplantation for patients in regional, rural, and remote areas, recommendations were made by the study participants and/or authors in most instances. A summary of the most prevalent barriers identified along with the recommended strategies to address them are presented in Fig. 2. Increased education and awareness around transplantation processes as well as both living and deceased organ donation was a recurrent theme amongst the included studies [35, 36, 39, 42, 45, 48]. A recently conducted review highlighted three important priorities when developing pre-transplant education: flexibility in the way in which education is delivered, involvement of peers with experiential knowledge, and tailoring the education for needs of vulnerable or marginalised populations [60]. Video, telehealth, or web-based programs focusing on increased education around kidney transplantation have been implemented or trialled, and some tailored to target particular ethnic groups, with available results indicating good patient acceptance [61–63]. Interventions targeting education of both health care staff and patients within dialysis facilities have also been shown to increase rates of transplant referral [64].

Given that many of the identified barriers to kidney transplantation for rural and remote patients arise from the need to travel, providing telehealth services to improve access to transplant evaluation and work-up processes should also be considered. It has been shown that utilisation of telehealth services in the pre-transplant phase would reduce time and costs associated with travel for potential recipients and carers, reduce wait time to transplant evaluation, and reduce perceived barriers to referral by health professionals [65, 66].

Another recurrent recommendation made by both participants and authors of studies included in this review was the need for increased social support for potential transplant recipients and their carers, including more culturally appropriate services [33, 40, 41, 49, 50, 52]. Provision of both informational and emotional support from peer mentors with lived experience of CKD and transplant has been identified as an important tool to help patients and carers navigate the various barriers to kidney transplantation [60, 67, 68]. A recent systematic review looked at the different types of patient navigators (nurse, social worker, peer) and their various roles within the CKD setting, and it was shown that patient navigators improve the completion of steps required for kidney transplant work-up and waitlisting [69]. Further research is planned/underway to determine the effectiveness of these programs in reducing barriers to kidney transplantation for rural and remote and Indigenous populations specifically [52, 70].

### Limitations

The strengths of this review include the broad search strategy used as well as the focus on presenting perspectives of CKD patients, their caregivers and relevant health professionals to comprehensively identify barriers to kidney transplantation for rural and remote populations. However, all but one of the included studies were undertaken in either Australia, New Zealand, United States or Canada, which may limit the generalisability of the findings across other countries. Another limitation includes the lack of consistency around the definitions of the terms “regional”, “rural” and “remote” used across included studies, with many different classification methods used. As such, terms used in this review have been kept consistent with those used in the specific study or reference cited.

## Conclusions

The process of assessing and determining a patients’ suitability to receive a kidney transplantation is both complex and time consuming and even once eligibility is confirmed, time spent on the wait-list can be prolonged. This review shows that patients residing in regional, rural, and remote areas face many additional barriers to kidney transplantation, which are primarily associated with the need to travel or relocate to metropolitan areas, where medical testing and/or transplantation facilities are located. It also offers a novel insight into the different health priorities between patient/carer and health professional populations, and highlights the need for a multifaceted approach when developing interventions to overcome identified barriers, to ensure the needs of both populations are met. There is a need for further research into how the inequity of access to kidney transplantation for this patient population can be resolved, and reviewing the literature to identify and describe identified barriers across studies may inform strategies to address this.

## Supplementary Information

Below is the link to the electronic supplementary material.

Supplementary file1 (PDF 53 KB)

Supplementary file2 (PDF 131 KB)

## Data Availability

No new data was collected as part of this review.
