# Supplementary material for: Identifying the barriers to kidney transplantation for patients in rural and remote areas: a scoping review
Source: J Nephrol. 2023 Sep 1;37(6):1435–47. doi: 10.1007/s40620-023-01755-0 (PMC11473485; doi:10.1007/s40620-023-01755-0)
Supplement: Supplementary file 1 — Supplementary file1 (PDF 53 KB) [file 40620_2023_1755_MOESM1_ESM.pdf]

Identifying the barriers to kidney transplantation for patients in rural and remotes areas – A scoping review

Tara K Watters, BPharm(Hons),[1,2] Beverley D Glass, PhD,[1] Andrew J Mallett, PhD,[1,3,4]

[1]*College of Medicine & Dentistry, James Cook University, Townsville, QLD, Australia*
[2]*Department of Renal Medicine, Cairns Hospital, Cairns, QLD, Australia*
[3]*Department of Renal Medicine, Townsville University Hospital, Townsville, QLD, Australia*
[4]*Institute for Molecular Bioscience, The University of Queensland, Brisbane, QLD, Australia*

Correspondence: Tara K Watters tara.watters@my.jcu.edu.au

Online Resource 1 – Database search strategies

All database searches were originally conducted on July 21, 2022, and then updated on December 20, 2022 to identify new studies that met the study criteria

MEDLINE (Ovid)

#	Searches	Results
1	Kidney Transplantation/	102,865
2	Rural Health/ or Rural Population/ or Hospitals, Rural/ or Rural Nursing/ or exp Rural Health Services/	103,788
3	Indigenous Peoples/ or Health Services, Indigenous/ or exp Australia/	166,670
4	2 or 3	264,229
5	1 and 4	513

CINAHL Complete

#	Searches	Results
S1	(MH "Kidney Transplantation+")	12,462
S2	(MH "Rural Health Personnel") OR (MH "Rural Health Centers") OR (MH "Hospitals, Rural") OR (MH "Rural Population") OR (MH "Rural Health Services") OR (MH "Rural Health Nursing") OR (MH "Rural Health") OR (MH "Rural Areas")	53,876
S3	(MH "Australia+")	129,539
S4	(MH "Indigenous Peoples+") OR (MH "Indigenous Health") OR (MH "Health Services, Indigenous")	24,074
S5	S2 OR S3 OR S4	195,044
S6	S1 AND S5	223

Emcare on Ovid

#	Searches	Results
1	exp kidney transplantation/	16,459
2	rural population/ or rural health nursing/ or urban rural difference/ or exp rural health care/ or rural health/ or rural hospital/ or rural area/ or rural hygiene/	47,020
3	exp "Australia and New Zealand"/ or exp Australia/	81,967
4	exp indigenous health care/ or exp Indigenous Australian/ or exp indigenous people/	14,521
5	2 or 3 or 4	138,299
6	1 and 5	193

Scopus

#	Searches	Results
1	((TITLE-ABS-KEY ("kidney transplant" OR "kidney grafting" OR "kidney transplantation" OR "kidney transplantations" OR "renal transplantation" OR "renal transplantations" OR "kidney graf" OR "transplantation")) AND ((TITLE-ABS-KEY ("rural population" OR "rural health" OR "rural health care" OR "rural area" OR "rural areas" OR "rural hospital" OR "rural hospitals" OR "rural health nursing" OR "rural health services" OR "rural nursing" OR "rural health centers")) OR (TITLE-ABS-KEY ("australia" OR "australia and new zealand" OR "indigenous health services" OR "indigenous people" OR "indigenous peoples" OR "indigenous health" OR "indigenous health care" OR "indigenous australian")))) AND (TITLE-ABS-KEY (barrier* OR "health services accessibility" OR "health care disparities' OR inequit* OR limitation* OR access))	493
