# Supplementary material for: Identifying the barriers to kidney transplantation for patients in rural and remote areas: a scoping review
Source: J Nephrol. 2023 Sep 1;37(6):1435–47. doi: 10.1007/s40620-023-01755-0 (PMC11473485; doi:10.1007/s40620-023-01755-0)
Supplement: Supplementary file 2 — Supplementary file2 (PDF 131 KB) [file 40620_2023_1755_MOESM2_ESM.pdf]

Identifying the barriers to kidney transplantation for patients in rural and remotes areas – A scoping review

Tara K Watters, BPharm(Hons),[1,2] Beverley D Glass, PhD,[1] Andrew J Mallett, PhD,[1,3,4]

[1]*College of Medicine & Dentistry, James Cook University, Townsville, QLD, Australia*
[2]*Department of Renal Medicine, Cairns Hospital, Cairns, QLD, Australia*
[3]*Department of Renal Medicine, Townsville University Hospital, Townsville, QLD, Australia*
[4]*Institute for Molecular Bioscience, The University of Queensland, Brisbane, QLD, Australia*

Correspondence: Tara K Watters tara.watters@my.jcu.edu.au

Online Resource 2 – Summary of included studies

**Author(s)<br>Year<br>Origin**	**Aims / Purpose**	**Methodology<br>Population & Sample Size<br>Validation of Data Collection Methods (if stated)**	**Key Themes Identified**	**Recommendations by Authors**
**QUANTITATIVE STUDIES**				
Barnieh et al<br><br>2018<br><br>Australia	To describe the direct and indirect costs incurred by Australian living kidney donors	Postal survey<br><br>Living kidney donors (n = 49)	Burden of travel & distance from treatment<br><br>SOCIAL & CULTURAL ISSUES<br>> Financial burden of treatment	To implement tailored reimbursement programmes to reduce the economic consequences of living donation, that take into consideration the type of donor instead of creating a 'one size fits all' approach to the financial burden living donors may face
Bartolomeo et al<br><br>2019<br><br>United States	To examine nephrologists' perceptions about factors that are important in excluding patients from KT referral	Survey<br><br>Nephrologists from both rural (n = 63) and urban (n = 163) regions	Perceived limited understanding of illness<br><br>Burden of travel & distance from treatment<br><br>SOCIAL & CULTURAL ISSUES<br>> Perceived social & cultural issues<br><br>SYSTEM-LEVEL FACTORS<br>> System-level factors as barriers	To improve availability of transplant-related education targeting nephrologists practicing in rural areas and exploring the potentials of telemedicine to bridge the rural-urban gap in perceptions of transplant candidacy<br><br>To conduct further research to define social support, to improve tools for assessment of social support and to study the impact of social support on transplant outcomes in order to optimise patient referral for transplantation

Cass et al<br><br>2007<br><br>Australia	To elucidate the factors affecting nephrologists' decision-making on patients' suitability for kidney transplantation	Postal survey<br><br>Australian nephrologists and nephrology trainees (n = 183)	COMPLIANCE ISSUES<br>> Pre-transplant compliance & engagement<br><br>TRANSPLANTATION PROCESSES<br>> Medical comorbidities as a barrier to transplantation<br><br>SYSTEM-LEVEL FACTORS<br>> System-level factors as barriers	To review weight accorded to the competing principles of potential benefit for an individual patient vs maximizing health benefit for the community in kidney transplantation
Cunningham et al<br><br>2006<br><br>Australia	To explore the attitudes and perceptions of Australian nephrologists towards LKD	Postal survey<br><br>Australian nephrologists and nephrology trainees (n = 184)	Fear of negative outcomes<br><br>PHYSICAL & PSYCHOSOCIAL WELLBEING<br>> Safeguarding psychological wellbeing<br>> Justifying LKD sacrifice	To increase the availability of kidneys from deceased donors, through campaigns to increase public awareness of kidney disease and acceptance of organ donation<br><br>To implement a systematic approach to the collection of long-term outcome data concerning living donors, so potential risks are better understood by clinicians
Weiland et al<br><br>2013<br><br>Australia	To determine Australian emergency department clinicians' cultural and religious barriers to organ and tissue donation (OTD)	Cross sectional survey (with some summarised qualitative data)<br><br>Nurses and physicians working in Australian emergency departments (n = 648)	Communication barriers<br><br>SOCIAL & CULTURAL ISSUES<br>> Perceived social & cultural issues<br><br>TRANSPLANTATION PROCESSES<br>> Barriers to facilitating OTD	To develop targeted education delivered in clinical and religious forums in order to minimise cultural and religious barriers to OTD for health professionals
**QUALITATIVE STUDIES**				
Anderson et al<br><br>2009<br><br>Canada	To explore the views of Canadian kidney health professionals on the barriers facing Aboriginal ESKD patients accessing kidney transplants	In-depth semi-structured interviews<br><br>Senior transplanting nephrologists and programme directors (n = 8), transplant managers and coordinators (n = 5), clinical nurse specialists and patient educators (n = 8) and Aboriginal renal nurses (n = 2)	Burden of travel & distance from treatment<br><br>TRANSPLANTATION PROCESSES<br>> Shortage of donor kidneys<br><br>SOCIAL & CULTURAL ISSUES<br>> Perceived social & cultural issues<br><br>SYSTEM-LEVEL FACTORS<br>> System-level factors as barriers	Nil

Anderson et al<br><br>2012<br><br>Australia	To investigate: whether Indigenous patients are commonly characterised as ‘non-compliers’; how estimations of patient compliance factor into Australian nephrologists’ decision-making about transplant referral; and whether this may pose a particular barrier for Indigenous patients accessing transplants **(part of the IMPAKT study)**	In-depth semi-structured interviews<br><br>Australian nephrologists (n = 19)<br><br>Interview schedule piloted with 2 nephrologists; use of "peer interviewing" to improve quality of data collected; interview transcripts were returned to participants (if requested at time of interview) with invitation to amend if necessary	Fear of negative outcomes<br><br>Burden of travel & distance from treatment<br><br>TRANSPLANTATION PROCESSES<br>> Shortage of donor kidneys<br>> Balancing benefit to patient VS maximising utility of donor kidneys<br><br>COMPLIANCE ISSUES<br>> Poor definition & assessment of compliance<br>> Pre-transplant compliance & engagement<br><br>SOCIAL & CULTURAL ISSUES<br>> Perceived social & cultural issues	To prioritise development of a transparent and fair system for patient selection as a key objective of organ transplantation programs, for example development of an agreed national approach to patient selection and organ distribution<br><br>To investigate particular selection criteria to ascertain their relevance and validity in determining patient suitability for transplant
Anderson et al<br><br>2012<br><br>Australia	To understand how the demands of dialysis impact on, and are impacted by, the lives of Indigenous patients **(part of the IMPAKT study)**	In-depth semi-structured interviews<br><br>Indigenous (n = 146) and non-Indigenous (n = 95) ESKD patients undergoing either HD, PD or Tx who commenced RRT within last 5 years<br><br>Use of interpreters (where required) and "peer interviewing" to improve quality of data collected; interview transcripts were returned to participants (if requested at time of interview) with invitation to amend if necessary	Communication barriers<br><br>Limited understanding of illness or treatment options<br><br>SYSTEM-LEVEL FACTORS<br>> Impact of late presentation or diagnosis<br><br>SOCIAL & CULTURAL ISSUES<br>> Indigenous specific cultural responsibilities<br>> Dislocation from family and support network<br><br>PHYSICAL & PSYCHOSOCIAL WELLBEING<br>> Physical and psychosocial effects of treatment<br><br>COMPLIANCE ISSUES<br>> Non-compliance or inability to engage with treatment	To establish improved linkages between specialist renal services and primary care in regional settings to support patients to better prepare, and improve communication and patient education to strengthen patients' understanding of their situation<br><br>To review the weight placed on patient “compliance” in decision-making, and transplant and home dialysis programs to establish more systematic, transparent approaches to “compliance” in their guidelines

Anderson et al<br><br>2013<br><br>Australia	1) How, and how effectively, are Indigenous ESKD patients informed and educated about their illness and treatment options, including transplant?<br>2) What factors, processes and conditions shape decision- making in relation to transplant options for ESKD patients, in particular for Indigenous patients?<br>3) What barriers prevent Indigenous ESKD patients receiving transplants at rates comparable to their non-Indigenous counterparts?	In-depth semi-structured interviews<br><br>Indigenous (n = 146) and non-Indigenous (n = 95) ESKD patients undergoing either HD, PD or Tx who commenced RRT within last 5 years<br><br>Use of interpreters (where required) and "peer interviewing" to improve quality of data collected; interview transcripts were returned to participants (if requested at time of interview) with invitation to amend if necessary	Limited understanding of illness or treatment options<br><br>Communication barriers<br><br>SOCIAL & CULTURAL ISSUES<br>> Indigenous specific cultural responsibilities<br>> Dislocation from family & support network<br><br>COMPLIANCE ISSUES<br>> Non-compliance or inability to engage with treatment<br><br>PHYSICAL & PSYCHOSOCIAL WELLBEING<br>> Physical & psychosocial effects of treatment<br><br>SYSTEM-LEVEL FACTORS<br>> Impact of late presentation or diagnosis	To develop alternative service delivery models for Indigenous patients that are regionally specific<br><br>To establish more effective and appropriate communication and education for Indigenous kidney disease patients<br><br>To establish delivery of dialysis services closer to Indigenous patients' home communities, more cross-cultural training of health-care workers and clinicians, an increase in the use of interpreter services (particularly in Indigenous languages with greater numbers of patients), and training of more health-care providers from ethnic and racial minority backgrounds—particularly Indigenous Australians
Bennett et al<br><br>1995<br><br>Queensland (Australia)	To discuss folk and lay understandings of renal physiology and disease aetiology, and social and cultural factors in dialysis and transplantation, in a group of Aboriginal and Torres Strait Islander renal transplant recipients	Extended flexible narrative-style interviews<br><br>Aboriginal and Torres Strait Islander cadaveric-transplant recipients and dialysis patients (n = 11), and health professionals from a major referral hospital (sample size not stated)	Limited understanding of illness or treatment options<br><br>Fear of negative outcomes<br><br>Communication barriers<br><br>SOCIAL & CULTURAL ISSUES<br>> Indigenous specific cultural responsibilities<br>> Experiences of racism or cultural bias<br>> Religion, spirituality & cultural beliefs<br>> Perceived social & cultural issues<br>> Involvement of family & community in treatment decisions<br>> Dislocation from family & support network<br><br>TRANSPLANTATION PROCESSES<br>> Perceptions around organ donation<br>> Hesitancy to accept a donated kidney<br><br>COMPLIANCE ISSUES<br>> Non-compliance or inability to engage with treatment<br><br>PHYSICAL & PSYCHOSOCIAL WELLBEING<br>> Physical & psychosocial effects of treatment<br><br>MOTIVATION FOR TRANSPLANTATION<br>> Motivation for transplantation	To develop culturally appropriate support systems and develop improved, comprehensible education information

Devitt et al<br><br>2017<br><br>Australia	To explore Indigenous ESKD patients' views on transplantation as a treatment option **(part of the IMPAKT study)**	Extended flexible narrative-style interviews<br><br>Adult Indigenous ESKD patients (n = 146)<br><br>Use of interpreters (where required) and "peer interviewing" to improve quality of data collected; interview transcripts were returned to participants (if requested at time of interview) with invitation to amend if necessary	Communication barriers<br><br>Fear of negative outcomes<br><br>Limited understanding of illness or treatment options<br><br>SOCIAL & CULTURAL ISSUES<br>> Dislocation from family & support network<br>> Involvement of family & community in treatment decisions<br>> Religion, spirituality & cultural beliefs<br><br>PHYSICAL & PSYCHOSOCIAL WELLBEING<br>> Physical & psychosocial effects of treatment<br><br>MOTIVATION FOR TRANSPLANT<br>> Motivation for transplant<br><br>TRANSPLANTATION PROCESSES<br>> Hesitancy to accept a donated kidney	To establish collaboration between transplant units and local treatment providers to develop user-friendly, culturally informed and region-specific patient education programs and practices for Indigenous ESKD/transplant patients<br><br>To develop a better understanding of how (or if) factors related to patient, service, health system and broader social determinants predict transplant outcomes<br><br>To review current utilitarian approach to organ allocation with respect to equity of access, as it may be more appropriate to compare the risks and benefits of transplant versus remaining on dialysis for Indigenous patients
Ghahramani et al<br><br>2014<br><br>United States	To explore different perceptions of rural and urban patients with CKD about kidney transplant	Focus group discussions<br><br>Adult patients with stage 5 CKD or ESKD (n = 23)<br><br>Topic guide based on literature review and peer discussion; the guide was revised following pilot testing among seven dialysis patients at the principal investigator's institution	Burden of travel & distance from treatment<br><br>Limited understanding of illness or treatment options<br><br>SOCIAL & CULTURAL ISSUES<br>> Religion, spirituality & cultural beliefs<br><br>TRANSPLANTATION PROCESSES<br>> Tedious pre-transplant work-up<br>> Hesitancy to accept a donated kidney<br><br>MOTIVATION FOR TRANSPLANT<br>> Motivation for transplant	To develop a better awareness of beliefs, concerns and fears of patients amongst physicians when discussing the option of transplant

Ghahramani et al<br><br>2014<br><br>United States	To explore different perceptions of urban and rural nephrologists regarding patient suitability for transplant	Focus group discussions<br><br>Nephrologists involved in the care of at least 20 patients with ESRD, practicing in either a rural (n = 9) or urban (n = 11) setting<br><br>Topic guide developed based on investigator discussions and literature review, modified based on pilot testing according to 2 nephrology fellows and 2 nephrologists	Burden of travel & distance from treatment<br><br>Communication barriers<br><br>Fear of negative outcomes<br><br>Perceived limited understanding of illness<br><br>SOCIAL & CULTURAL ISSUES<br>> Perceived social & cultural issues<br><br>TRANSPLANTATION PROCESSES<br>> Medical comorbidities as a barrier to transplantation<br>> Balancing benefit to patient VS maximising utility of donor kidneys<br>> Shortage of donor kidneys	Nil
Hanson et al<br><br>2016<br><br>Australia & New Zealand	To describe nephrologists' attitudes towards recipient eligibility and access to living kidney donor transplantation	Semi-structured interviews<br><br>Nephrologists involved in referral and assessment for living donor transplantation in Australia and New Zealand (n = 41)<br><br>Interview guide based on a systematic literature review of disparities in kidney transplantation and discussion among the research team	Burden of travel & distance from treatment<br><br>Communication barriers<br><br>Fear of negative outcomes<br><br>Perceived limited understanding of illness<br><br>SOCIAL & CULTURAL ISSUES<br>> Perceived social & cultural issues<br><br>COMPLIANCE ISSUES<br>> Poor definition & assessment of compliance<br>> Pre-transplant compliance & engagement<br><br>TRANSPLANTATION PROCESSES<br>> Shortage of donor kidneys<br>> Medical comorbidities as a barrier to transplantation<br>> Balancing benefit to patient VS maximising utility of donor kidneys<br><br>MOTIVATION FOR TRANSPLANT<br>> Advocating for transplant as a treatment option<br><br>PHYSICAL & PSYCHOSOCIAL WELLBEING<br>> Safeguarding psychological wellbeing<br>> Justifying LKD sacrifice<br><br>SYSTEM-LEVEL FACTORS<br>> System-level factors as barriers	To implement an explicit pathway for patient education and referral to transplant services to promote consistent and equitable recommendations to patients<br><br>To develop evidence-based recommendations to address psychosocial risk factors, including nonadherence, depression, and low social support<br><br>To implement a process in which both patient outcomes and transplant volume are regarded as equally important in quality evaluations to ensure that efforts to maximize outcomes are not maintained at the expense of expanding access to transplantation

Kelly et al<br><br>2022<br><br>South Australia (Australia)	To describe the experiences, perceptions and suggested improvements in healthcare identified by Aboriginal patients, families and community members living with kidney disease in South Australia	Semi-structured focus groups (consultation methodology)<br><br>Aboriginal kidney care patients, family members, other community members who are carers (n = 46)<br><br>All butcher's paper and group facilitation notes were transcribed written into site specific reports, checked by reference group and approved by participants in location to ensure accuracy, truth telling and community control	Burden of travel & distance from treatment<br><br>Communication barriers<br><br>Fear of negative outcomes<br><br>Limited understanding of illness or treatment options<br><br>SOCIAL & CULTURAL ISSUES<br>> Religion, spirituality & cultural beliefs<br>> Indigenous specific cultural responsibilities<br>> Dislocation from family & support network<br>> Involvement of family & community in treatment decisions<br><br>MOTIVATION FOR TRANSPLANT<br>> Motivation for transplant<br><br>TRANSPLANTATION PROCESSES<br>> Tedious pe-transplant work-up<br><br>SYSTEM-LEVEL FACTORS<br>> Impact of late presentation or diagnosis<br><br>PHYSICAL & PSYCHOSOCIAL WELLBEING<br>> Physical & psychosocial effects of treatment	Recommendations / strategies provided by participants as part of the study results, nil further recommendations made by authors
McGrath et al<br><br>2012<br><br>Queensland (Australia)	To explore the financial impact on the live renal donor in terms of testing, hospitalisation and surgery for kidney removal	Semi-structured interviews<br><br>Living kidney donors (n = 20)	Burden of travel & distance from treatment<br><br>SOCIAL & CULTURAL ISSUES<br>> Financial burden of treatment	To review current health policy and service delivery considerations to ensure financial costs do not continue to present a barrier to voluntary donation, and that potential donors are supported appropriately by the healthcare system
Misra et al<br><br>2021<br><br>India	To understand the beliefs and knowledge of a rural community toward organ donation and the identification of barriers for organ donation	Focus group discussions<br><br>Persons aged 18 years and above and residing in villages under PHC Dayalpur for at least 6 months (n = 48)<br><br>Topic guide developed based on issues identified from previous literature	Limited understanding of illness or treatment options<br><br>SOCIAL & CULTURAL ISSUES<br>> Religion, spirituality & cultural beliefs<br><br>TRANSPLANTATION PROCESSES<br>> Perceptions around organ donation	To address lack of awareness and knowledge around organ donation processes through use of campaigns and awareness programs

Scholes-Robertson et al<br><br>2022<br><br>Australia	To describe the experiences of caregivers of patients with kidney failure from rural Australian communities in accessing kidney replacement therapy	Semi-structured interviews<br><br>Adult caregivers of Australian rural patients with kidney failure treated with dialysis or kidney transplantation (n = 18)<br><br>Interview guide based on a literature review of patient and caregivers' perspectives on access to KRT in rural communities and discussion among the research team; investigator triangulation and member-checking helped ensure the findings reflected the full range and depth of the data	Burden of travel & distance from treatment<br><br>Communication barriers<br><br>Fear of negative outcomes<br><br>Limited understanding of illness or treatment options<br><br>SOCIAL & CULTURAL ISSUES<br>> Financial burden of treatment<br>> Dislocation from family & support network<br><br>SYSTEM-LEVEL FACTORS<br>> Lack of continuity of care<br><br>PHYSICAL & PSYCHOSOCIAL WELLBEING<br>> Physical & psychosocial effects of treatment	To implement interventions that address challenges highlighted such as navigation of complex health care services, travel, and accommodation; to minimize the ongoing financial strain; and to improve availability of psychosocial support and respite services<br><br>To consider measures such as support groups or rural patient navigator programs to assist with accessing medical and psychosocial services
Scholes-Robertson et al<br><br>2022<br><br>Australia	To describe clinicians' perspectives of equity of access to dialysis and kidney transplantation in rural areas	Semi-structured interviews<br><br>Nephrologists, nurses and social workers involved in the care of rural patients with CKD in Australia (n = 28)<br><br>Interview guide based on a literature review of patient and clinician perspectives on access to kidney replacement therapy and discussion among the research team; preliminary findings were sent to participants and their feedback was integrated into the final analysis	Burden of travel & distance from treatment<br><br>Communication barriers<br><br>Perceived limited understanding of illness<br><br>PHYSICAL & PSYCHOSOCIAL WELLBEING<br>> Safeguarding psychological wellbeing<br><br>SYSTEM-LEVEL FACTORS<br>> System-level factors as barriers<br><br>SOCIAL & CULTURAL ISSUES<br>> Perceived social & cultural issues	To develop strategies to improve access to kidney replacement therapy for patient in rural communities, through decreasing travel burden, minimisation of relocation and reducing out of pocket expenses of the patients<br><br>To implement patient navigator roles to assist with coordination of care, health literacy and CKD education support, practical support to assist with accommodation and transport issues, particularly in those with little or no social support

Scholes-Robertson et al<br><br>2022<br><br>Australia	To describe the perspectives of patients from rural communities on access to all forms of kidney replacement therapy	Semi-structured interviews<br><br>Rural patients with CKD aged >18 years in Australia (n = 28)<br><br>Interview guide based on a literature review of patient' and caregivers' perspectives on access to kidney replacement therapy in rural communities and discussion among the research team; investigator triangulation and member checking helped ensure the findings reflected the full range and depth of the data	Burden of travel & distance from treatment<br>Fear of negative outcomes<br><br>MOTIVATION FOR TRANSPLANT<br>> Motivation for transplant<br><br>TRANSPLANTATION PROCESSES<br>> Tedious pre-transplant work-up<br><br>SYSTEM-LEVEL FACTORS<br>> Lack of continuity of care<br><br>SOCIAL & CULTURAL ISSUES<br>> Dislocation from family & support network<br>> Financial burden of treatment<br><br>PHYSICAL & PSYCHOSOCIAL WELLBEING<br>> Physical & psychosocial effects of treatment	Recommendations / strategies provided by participants as part of the study results, nil further recommendations made by authors
Scholes-Robertson et al<br><br>2022<br><br>Australia	To ascertain participants' perspectives on barriers to access to dialysis and transplantation, to identify and prioritize the roles of a rural patient navigator, to discuss the acceptability and feasibility of implementing this role and identify possible outcomes that could be used to measure the success of the programme in a clinical trial	Workshop discussions<br><br>Rural dialysis/transplant patients, caregivers and health professionals (n = 42)<br><br>Questions based on a literature review of rural patient and care partners' perspectives of access to dialysis and transplantation, a systematic review of patient navigator programme and discussion among the investigator team; initial themes developed discussed with and agreed by all workshop facilitators	Burden of travel & distance from treatment<br><br>Communication barriers<br><br>Limited understanding of illness or treatment options<br><br>SOCIAL & CULTURAL ISSUES<br>> Involvement of family & community in treatment decisions<br>> Financial burden of treatment<br>> Dislocation from family & support network<br>> Religion, spirituality & cultural beliefs<br><br>PHYSICAL & PSYCHOSOCIAL WELLBEING<br>> Physical & psychosocial effects of treatment<br><br>SYSTEM-LEVEL FACTORS<br>> Lack of continuity of care	Recommendations / strategies provided by participants as part of the study results, nil further recommendations made by authors<br><br>Findings from this study to inform the co-design and implementation of a rural patient navigator programme for adults with CKD, with a particular focus on improving access to dialysis and transplantation

Tong et al<br><br>2011<br><br>Australia	To elicit nephrologists' perspectives on waitlisting patients for kidney transplant and the allocation of deceased kidneys	Semi-structured interviews<br><br>Practicing nephrologists in Australia who had a role in waitlisting patients or accepting deceased donor kidneys offered through the computer allocation algorithm (n = 25)<br><br>Interview guide developed based on a literature review on waitlisting and allocation of deceased donor kidneys, current waitlisting and allocation protocols, and discussion in the research team; questions were piloted with 2 nephrologists	Fear of negative outcomes<br><br>PHYSICAL & PSYCHOSOCIAL WELLBEING<br>> Safeguarding psychological wellbeing<br><br>TRANSPLANTATION PROCESSES<br>> Medical co-morbidities as a barrier to transplantation<br>> Balancing benefit to patient VS maximising utility of donor kidneys<br><br>COMPLIANCE ISSUES<br>> Pre-transplant compliance & engagement<br><br>SOCIAL & CULTURAL ISSUES<br>> Perceived social & cultural issues<br><br>SYSTEM-LEVEL FACTORS<br>> System-level factors as barriers	To augment current guidelines with recommendations underpinned by research on patient and community preferences for kidney transplant and a framework for eliciting patient and community preferences for waitlisting and allocation<br><br>To ensure transplant policy makers and organ procurement and allocation organisations are more explicit and accountable about the appropriate balance between maximising benefits and maintaining equity in different settings
Walker et al<br><br>2022<br><br>New Zealand	To explore the experiences and perceptions of rural and remote patients and families in relation to accessing health services for kidney disease in Aotearoa New Zealand	In-depth semi-structured interviews<br><br>Adult CKD patients and their caregivers who lived further than 100 km (62 miles) or more than 1 h drive from their nearest dialysis or transplant centre (n = 35)<br><br>Interview guide developed based on previous research in this area; initial themes identified were sent back to consenting participants to ensure consistency in capturing the themes and experiences	Burden of travel & distance from treatment<br><br>Communication barriers<br><br>SOCIAL & CULTURAL ISSUES<br>> Financial burden of treatment<br>> Dislocation from family & support network<br><br>TRANSPLANTATION PROCESSES<br>> Tedious pre-transplant work-up<br><br>COMPLIANCE ISSUES<br>> Non-compliance or inability to engage with treatment<br><br>SYSTEM-LEVEL FACTORS<br>> Lack of continuity of care<br><br>PHYSICAL & PSYCHOSOCIAL WELLBEING<br>> Physical & psychosocial effects of treatment	To develop rural services such as remote dialysis and kidney transplantation models of care, including community dialysis houses and augmented primary care in remote communities<br><br>To design health services and policy in a way that address the financial burden and poor communication related to financial entitlements

Walker et al<br><br>2022<br><br>New Zealand	To report on perceptions and experiences of prejudice and racism by indigenous Māori with kidney disease and their family members and donors	Semi-structured interviews<br><br>Māori patients with kidney disease who had considered, were being worked up for or who had already received a kidney transplant as well as family members and potential or previous donors (n = 40)<br><br>The interview adhered strictly to Kaupapa Māori principles to facilitate mutual understanding and building of relationships, connections and trust; some interviews were conducted by both interviewers to ensure consistency of interviewing	Communication barriers<br><br>Limited understanding of illness or treatment options<br><br>TRANSPLANTATION PROCESSES<br>> Tedious pre-transplant work-up<br>> Hesitancy to accept a donated kidney<br><br>COMPLIANCE ISSUES<br>> Non-compliance or inability to engage with treatment<br><br>SOCIAL & CULTURAL ISSUES<br>> Dislocation from family & support network<br>> Religion, spirituality & cultural beliefs<br>> Experiences of racism or cultural bias<br><br>PHYSICAL & PSYCHOSOCIAL WELLBEING<br>> Physical & psychosocial effects of treatment	Recommendations / strategies provided by participants as part of the study results, nil further recommendations made by authors
